# An analytic and systematic framework for estimating metabolic flux ratios from ^13^*C *tracer experiments

**DOI:** 10.1186/1471-2105-9-266

**Published:** 2008-06-06

**Authors:** Ari Rantanen, Juho Rousu, Paula Jouhten, Nicola Zamboni, Hannu Maaheimo, Esko Ukkonen

**Affiliations:** 1 Department of Computer Science, University of Helsinki, Finland; 2 Institute of Molecular Systems Biology, ETH Zurich, Switzerland; 3 VTT Technical Research Centre of Finland, Finland

## Abstract

**Background:**

Metabolic fluxes provide invaluable insight on the integrated response of a cell to environmental stimuli or genetic modifications. Current computational methods for estimating the metabolic fluxes from ^13^*C *isotopomer measurement data rely either on manual derivation of analytic equations constraining the fluxes or on the numerical solution of a highly nonlinear system of isotopomer balance equations. In the first approach, analytic equations have to be tediously derived for each organism, substrate or labelling pattern, while in the second approach, the global nature of an optimum solution is difficult to prove and comprehensive measurements of external fluxes to augment the ^13^*C *isotopomer data are typically needed.

**Results:**

We present a novel analytic framework for estimating metabolic flux ratios in the cell from ^13^*C *isotopomer measurement data. In the presented framework, equation systems constraining the fluxes are derived automatically from the model of the metabolism of an organism. The framework is designed to be applicable with all metabolic network topologies, ^13^*C *isotopomer measurement techniques, substrates and substrate labelling patterns.

By analyzing nuclear magnetic resonance (NMR) and mass spectrometry (MS) measurement data obtained from the experiments on glucose with the model micro-organisms *Bacillus subtilis *and *Saccharomyces cerevisiae *we show that our framework is able to automatically produce the flux ratios discovered so far by the domain experts with tedious manual analysis. Furthermore, we show by *in silico *calculability analysis that our framework can rapidly produce flux ratio equations – as well as predict when the flux ratios are unobtainable by linear means – also for substrates not related to glucose.

**Conclusion:**

The core of ^13^*C *metabolic flux analysis framework introduced in this article constitutes of flow and independence analysis of metabolic fragments and techniques for manipulating isotopomer measurements with vector space techniques. These methods facilitate efficient, analytic computation of the ratios between the fluxes of pathways that converge to a common junction metabolite. The framework can been seen as a generalization and formalization of existing tradition for computing metabolic flux ratios where equations constraining flux ratios are manually derived, usually without explicitly showing the formal proofs of the validity of the equations.

## Background

From microorganisms to animals and plants, cells adjust their metabolic operations to fulfill the demand of energy and biosynthetic precursors. In nature this is a challenging task because substrate availability is typically limited and often changing in its composition. To ensure viability on a broad palette of chemically heterogeneous substrates, cells have developed intertwined enzymatic networks that also confer robustness against genetic mutations. Optimum redistribution of molecular fluxes in metabolism is achieved by multilevel regulation circuits. In recent years, experimental measurement of in vivo metabolic fluxes has attracted much attention. For example, in biotechnology metabolic fluxes are utilized to lead rational strain engineering, whereas systems biologists assess fluxes to unravel targets and mechanisms of metabolic regulation.

Metabolic fluxes are often estimated using flux balance analysis (FBA) [1,2]. In FBA, fluxes are solved by fixing some objective for the metabolism of an organism, such as maximal growth. Then, a corresponding optimization problem is solved by using the stoichiometry of the metabolic network as a constraint to the optimization. FBA is a viable method for studying the metabolic capabilities of an organism, but as a method for estimating metabolic fluxes it has some weaknesses. First, selecting the correct objective for the metabolism is far from trivial [3], especially when mutant strains or behaviour in exceptional environmental conditions is analyzed. Second, there can be many biologically interesting flux distributions that give an optimal solution to the optimization problem of FBA.

A more direct method for experimental determination of the metabolic fluxes is to feed an organism with ^13^*C *labelled substrate, observe the fate of ^13^*C *atoms in the cell at isotopomeric steady state with mass spectrometry (MS) or nuclear magnetic resonance (NMR) measurements, and then infer the metabolic fluxes from the measurements. The rationale behind these ^13^*C *tracer experiment is that, often alternative pathways between metabolites in the network manipulate the carbon backbones of the metabolites differently, thus inducing different ^13^*C *labelling patterns to metabolites. Then, constraints to fluxes complementary to the basic stoichiometric constraints can be derived by measuring the relative abundances of different labelling patterns in the metabolites.

The main difficulty in applying the procedure in practice is that current measurement techniques only can produce incomplete information about relative abundances of different ^13^*C *labelling patterns, the *isotopomer distributions*, of some metabolites, usually protein bound amino acids in the network, and no isotopomer information at all for many intermediate metabolites of interest [4-6]. This imposes a highly non-linear dependency between the measured isotopomer distributions of the metabolites and the metabolic fluxes, which is very challenging to solve both computationally and statistically.

Currently, two main approaches for ^13^*C *metabolic flux analysis exist. In the *global isotopomer balancing *approach, the problem of estimating metabolic fluxes from the isotopomer measurements is formulated as a nonlinear optimization problem, where candidate flux distributions are iteratively generated until they fit well enough to the experimental ^13^*C *labelling patterns [7-11]. Global isotopomer balancing is a versatile approach that can be applied with all network topologies, substrate labelling distributions and with all measurement techniques – also in short time scales where isotopomeric steady state is not reached [12-14]. However, due to the nonlinearity of the problem, it is hard to make sure that the optimization has converged to a global optimum and that this optimum is unique [15]. Also, to apply the global isotopomer balancing approach successfully, one usually needs comprehensive information on the uptake and production rates of external metabolites, as well as about biomass composition of the cell. This information can be hard to obtain, especially in large-scale experiments where dozens to hundreds of mutants or different organisms are comparatively analyzed [16,17].

A metabolic flux ratio approach (METAFoR) [4,18,19] for ^13^*C *metabolic flux analysis has traditionally relied more on the expertise of a domain specialist than advanced computational techniques. In metabolic flux ratio analysis, the aim is to write linear equations constraining the *ratios *of fluxes producing the same metabolite. The equations are manually derived by domain experts, by careful (and tedious) inspection of metabolic networks. The motivation for the approach is that, in many cases, the knowledge about the flux ratios already offers enough information about the response of an organism to its environment.

The ratio of competing fluxes or pathways producing the same metabolite is easy to understand, and in many cases easier to estimate reliably than all the fluxes in the network – some interesting flux ratios might be obtainable from scarce measurement data or from the incomplete model of metabolic network that would not allow a reliable estimation of a complete flux distribution using global isotopomer balancing. Flux ratios can also be obtained without knowing the uptake and production rates of external metabolites of the biomass composition. And, if enough non-redundant flux ratios are identified, it is possible to use this information to construct and solve an equation system for the full flux distribution of the metabolic network [20-22].

As a downside, manually derived flux ratio equations depend heavily on the topology of a metabolic network, measurement capabilities and substrate labelling distributions. Thus, each time a new organism or new mixture of substrates are introduced, flux ratio equations have to be verified and new ones possibly derived. To date, flux ratio equations are manually derived for central carbon metabolisms of three model organisms on glucose, *S. cerevisiae *[17,23], *B. subtilis *[24] and for *Escherichia coli *[18,19]. Recently, flux ratio equations of *S. cerevisiae *were modified for *Pichia pastoris *grown on glycerol and on glycerol/methanol mixtures [25,26]. Facilitating the process of deriving flux ratio equations for other organisms and other substrates clearly calls for automatic tools. Also, many times the (simplifying) assumptions made by the expert in the derivation and solution of flux ratio equations, are not reported in detail. Thus, it is often nontrivial to verify the correctness of given flux ratio equations.

In this article we present a novel automatic framework for deriving flux ratios when the measurement data and the model of metabolic network are given as input. The framework is based on the graph algorithmic flow analysis of metabolite fragments in the metabolic network [27] and on the interpretation and manipulation of both NMR and MS data with vector space techniques [21]. The goal of our work is to combine the good aspects of global isotopomer balancing and manual flux ratio analysis: like global isotopomer balancing, our framework is systematic and can be applied with all network topologies, substrates and substrate labelling distributions and with all current isotopomer measurement techniques. Thus, laborious and error-prone manual inspection of metabolic network models and the tailoring of the equation systems constraining the fluxes separately for each experimental setting required in manual flux ratio techniques can be avoided. On the other hand, during the automated construction of flux ratios we resort to linear optimization techniques only, combined with graph algorithms of polynomial worst case time complexity. Thus, our framework is computationally efficient and avoids problems with local and multiple optima frequently met in global isotopomer balancing. The trade-off of this philosophy is, however, the requirement of measuring isotopomer distributions of metabolites more rigorously to obtain full flux distribution. Given insufficient measurements, our framework can solve the flux ratios only for some, but not necessary for all metabolites in the network. We expect that, especially as measuring isotopomers of intermediate metabolites becomes more routine, our framework will be an attractive method for ^13^*C *flux analysis.

## Results and Discussion

In this section we demonstrate the applicability of the presented framework by empirical results. We show that our automatic and systematic framework is able to reproduce flux ratios previously determined by a manual analysis from NMR and GC-MS isotopomer measurements of protein bound amino acids of *S. cerevisiae *and *B. subtilis *on glucose. Thus, we can conclude that the presented framework is powerful enough to provide interesting flux ratio information in the well studied experimental settings. Furthermore, we show that the framework can be applied to study less known experimental conditions without any further effort by discovering the flux ratios that can be estimated when *B. subtilis *is grown on malate instead of glucose. The results of this analysis show that our framework can detect profound effects the change of external substrate can have to the flux ratio computations. The results indicate that our framework is a good tool to study flux ratios of microbes in different experimental conditions – a claim that will try to validate with more experiments in our further work.

We obtained NMR and GC-MS labelling data, where isotopomer distributions of protein bound amino acids of *S. cerevisiae *and *B. subtilis *grown on different conditions were measured. Then, available flux ratios were computed with the presented framework. Models of metabolic networks applied in the analysis can be found from the supplementary material of this article: additional files 1 and 2 contain the SBML model file and a visualization of the model of *S. cerevisiae*, while additional files 3 and 4 contain the same information for *B. subtilis*. In the models, some simplifications common to ^13^*C *metabolic flux analysis were applied by pooling metabolites whose isotopomer pools can be assumed to be fully mixed (cf. [28]). Pooling of metabolites was carried for (i) the three pentose-phosphates in PPP, (ii) phopshotrioses between GA3P and PEP in glycolysis, and (iii) oxaloacetate and malate in the TCA cycle. In these cases, pooling is justified by the existence of fast equilibrating, bidirectional reactions between the listed intermediates and the empirical evidence that their isotopic labeling is not distinguishable with the current analytical tools. Cofactor metabolites were excluded from the model as cofactor specificities and activities are not accurately known for many reactions.

The bulk of the carbon mappings of reactions in the metabolic network were provided by ARM project [29]. Carbon mappings from amino acids to their precursors were conform to [4] and [23]. Before the analysis of the real measurement data, the correctness of the implementation of the framework was empirically verified by estimating flux ratios for junction metabolites in the metabolic network of *S. cerevisiae *from the artificial data generated by the 13C-FLUX software [8].

### NMR measurements from *S. cerevisae *on glucose

In the first experiment we analyzed NMR isotopomer measurement data from protein bound amino acids of *S. cerevisiae *that was grown on uniformly labelled glucose (see Section *Experimental NMR and GC-MS methods *for more details on experimental settings).

From the 15 measured amino acids we were able to estimate flux ratios for seven junction metabolites: oxaloacetate, PEP, glycine and serine on cytosol and for oxaloacetate, acetyl-CoA and pyruvate in mitochondria. Furthermore, an upper bound for a ratio of GA3P molecules that have visited the transketolase reaction was obtained by manually simplifying the model to imitate the previously reported ways to manually compute the corresponding upper bound (cf. [4]). (The structural analysis of the metabolic network model described in Section *Structural analysis of isotopomer systems *can help in discovering such simplifications, but they also need some expert insight. As the simplifications are currently not done automatically, the systematical framework is unable to validate them.)

The computed flux ratios were compared with the manually derived ratios [23], when the assumptions made in the manual derivation of flux ratios were consistent with the model used. In all cases, automatically computed flux ratios agreed well with the manually derived ratios (Table 1). Differences between the estimations can be explained by numerical instabilities and by differences in computational procedures: in manually derived ratios the estimations are based on the breakage of a single bond in different routes leading to a metabolite while in our framework more isotopomer information is possible utilized in the estimation.

**Table 1:** Estimated flux ratios from NMR measurements of *S. cerevisiae*.

flux ratios	our framework	METAFoR
PYR(mit) from MAL(mit) : PYR(mit) from PYR(cyt)	0.05 : 0.95	0.03 : 0.97
OAA(mit) from TCA-cycle : OAA(mit) from OAA(cyt)	0.50 : 0.50	0.50 : 0.50
PEP from OAA(cyt) : PEP from GA3P	0 : 1	0.04 : 0.96
OAA(cyt) from PYR(cyt) : OAA(cyt) from OAA(mit)	0.40 : 0.60	0.43 : 0.57
GLY from SER : GLY from C1 + CO2	0.96 : 0.04	0.96 : 0.04

Directly comparable flux ratios computed from the NMR data by the framework presented in this paper and by manual flux ratio analysis (METAFoR).

### GC-MS measurements from *B. subtilis *on glucose

In the second experiment we analyzed GC-MS isotopomer measurement data from protein bound amino acids of *Bacillus subtilis *that was grown on uniformly labelled glucose (see Section *Experimental NMR and GC-MS methods *for more details on experimental settings).

In comparison to eukaryotic *S. cerevisiae*, the metabolic network of prokaryotic *B. subtilis *lacks cellular compartments. Thus, from the point of view of ^13^*C *metabolic flux analysis, there are fewer interesting junction metabolites in the central carbon metabolism of *B. subtilis *where the flux ratios can be estimated. From the GC-MS measurements of 14 amino acids we were able to compute flux ratios for four junction metabolites – oxaloacetate, pyruvate, PEP and glycine – when [U-13C]-glucose was used as a carbon source. Furthermore, an upper bound for a ratio of GA3P molecules that have visited transketolase reaction was obtained by manually simplifying the model of the metabolic network. Excluding pyruvate, we were able to compute the same ratios with [1-13C]-glucose as a carbon source.

We compared the computed flux ratios to ones obtained with the software FiatFlux [30] that is based on the manually derived analytic equations for computing flux ratios. Currently, manually derived flux ratio equations for [1-13C]-glucose as a carbon source exist only for PEP and for the upper bound to the flux through oxidative pentose phosphate pathway. In general, the flux ratios computed with different methods from the same data and with the same assumptions about the topology of metabolic network were in good agreement (Table 2). (As a data cleaning procedure, we removed from [1-13C]-glucose data the mass distributions of fragments whose fractional enrichment deviated more than 5% from the assumed fractional enrichment of 20% in [U-13C]-data. This was done because differences in fractional enrichments can be tracked in uniformly labelled data where the fractional enrichment of each fragment is know *a priori*, but not in positionally labelled data, where the fractional enrichment of a fragment depends on the network topology and the fluxes. This kind of irregularities are in general caused by noise in fragments with low intensity or by coeluting analytes with overlapping fragment masses.) Again, differences between the flux ratios estimated by different methods can be explained by numerical instabilities and by differences in isotopomer information applied during the estimation. Variation in the estimated flux ratios between repeated experiments (six repetitions for [1-13C]-glucose experiment, four repetitions for uniformly labelled glucose experiment) was negligible.

**Table 2:** Estimated flux ratios from GC-MS measurements of *B. subtilis*.

flux ratios	our fw (UL)	our fw (1CL)	FiatFlux (UL)	FiatFlux (1CL)
PYR from MAL : PYR from PEP	0.01 : 0.99		0.04 : 0.96	
OAA from TCA-cycle : OAA from PYR	0.42 : 0.58	0.37 : 0.63	0.41 : 0.59	
PEP from OAA : PEP from GA3P	0 : 1	0 : 1	0.04 :0.96	0 : 1
GLY from SER : GLY from C1 + CO2	1 : 0	1 : 0	1 : 0	
SER from GLY : SER from GA3P	0.09 : 0.91		0.14 : 0.86	

Directly comparable flux ratios computed from the GC-MS data described by the framework presented in this paper (*our fw*) and by FiatFlux software [30]. *UL *denotes uniform labelling of external glucose, *1CL *external glucose labelled to the first carbon position.

### In silico calculability analysis of *B. subtilis *on malate

One of the strengths of the presented framework is that it is able to automatically produce metabolic flux ratios also when other external labelled substrates than commonly used glucose are fed to organisms. To demonstrate this ability, we applied our framework to predict what flux information would be available, if we feed *B. subtilis *with malate.

We applied the *in silico *calculability analysis (see Section *Calculability analysis*) to examine which flux ratios are calculable in the best case from GC-MS measurements of amino acids, when *B. subtilis *is grown on [U-13C]-labelled malate. Interestingly, our fragment flow analysis revealed that – with the reaction reversibilites in the applied model – the isotopomer distributions of GA3P, PEP and pyruvate depend only on the isotopomer distribution of the fragment containing the first three carbons of oxaloacetate, but not on the relative fluxes producing these metabolites. Thus, isotopomer balances for GA3P, PEP and pyruvate reduce to mass balances and the ratios of fluxes producing these metabolites cannot be estimated. This somewhat surprising phenomenon is due the fact that the rearrangements of carbon chains occurring in PPP pathway will affect only to the carbon fragments that will be recycled in the upper metabolism but not the carbon fragments that can flow back GA3P, PEP and pyruvate from PPP (we modelled a reaction from GA3P to F6P as unidirectional one).

Preliminary experiments with GC-MS data from *B. subtilis *grown on [U-13C]-labelled malate agreed with the results of fragment flow analysis: constraints to the isotopomer distributions of fluxes entering to these metabolites were identical within the limits of measurement accuracy. On the other hand, our framework was able to estimate for example the TCA-cycle activity also when *B. subtilis *is grown on malate, just as predicted by the calculability analysis.

## Conclusion

In this article we introduce a systematic and analytic framework for ^13^*C *metabolic flux ratio analysis. At the heart of the framework lie the techniques for flow analysis of a metabolic network and for manipulating isotopomer measurements as linear subspaces. These techniques facilitate the efficient and analytic computation of the ratios between the fluxes producing the same junction metabolite. The framework can be seen as a generalization and formalization of existing analytic methods for computing metabolic flux ratios [23,30,31] where equations constraining flux ratios are manually derived. Like the recent methods to improve the speed of the simulation of isotopomer distributions in the global isotopomer balancing framework [10,32], our framework relies on graph algorithms. However, both our goals and applied techniques are quite different from these approaches. In [10] and [32] connected components of isotopomer graphs are discovered to divide the simulation of isotopomer distributions to smaller subtasks. In our framework, flow analysis techniques are applied to discovered metabolite fragments with equivalent isotopomer distributions in every isotopomeric steady state.

Our experiments with NMR and MS data show that the framework is able to provide relevant information about metabolic fluxes, even when only constraints to the isotopomer distributions of protein-bound amino acids are measured.

Thanks to recent advancements in measurement technology improving the feasibility of mass isotopomer measurements of intermediate metabolites [13,33], we expect that the full power of the framework will be harnessed in near future. Measurements from intermediates will make it possible to use larger models of metabolic networks and estimate flux ratios more accurately, without simplifying assumptions about the topology of the metabolic network or directionality of the fluxes. However, these measurements will not be easy to conduct, because of the low abundances of intermediates in the cell. Thus, systematic methods for experimental planning and data quality control are required. The presented framework provides powerful tools for these tasks. First, the framework facilitates time saving *in silico *calculability analysis.

Second, the manipulation of isotopomer measurements as linear subspaces offers a natural way for comparing measurements from different metabolites that contain overlapping information to detect inconsistencies in the measurements: it is enough to compare propagated isotopomer information in the fragments that belong to the same equivalence class. Third, as MS isotopomer measurement techniques have to be developed separately for different intermediate metabolites or metabolite classes, it will be very useful to select a small subset of intermediates that gives enough information about the interesting metabolic fluxes with least experimental effort. In future research, we want to tackle this problem by generalizing our earlier experimental planning method [34], to all measurement data and to realistic measurement error models.

As the presented framework for ^13^*C *metabolic flux analysis only resorts to linear optimization techniques, it is not always able to provide as much information about the metabolic fluxes as the global isotopomer balancing frameworks utilizing more powerful, nonlinear optimization techniques [8,35], that do not necessarily converge to the global optimum. On the other hand, some flux ratios might be computable from the scarce data or incomplete model of metabolic network that does not allow global isotopomer balancing. The differences in the practical performance of different approaches require further research. We see these alternative approaches as complementary ones. A very nice goal would be an integration of our work with global isotopomer balancing: our analytic flux ratios could speed up and direct the optimization process of global isotopomer balancing, that would then fill in the flux ratios possibly missed by our framework.

## Methods

In this section we describe a systematic and analytic computational framework for ^13^*C *metabolic flux analysis. At the end of the section we also shortly describe the experimental method that were applied to produce the isotopomer measurement data that was analyzed in Section *Results and Discussion*.

The overall goal of our computational framework is to automatically infer from the available isotopomer measurement data produced by MS, MS-MS or NMR techniques, an equation system constraining the fluxes. The crucial question is to derive as many non-redundant equations as possible, ideally constraining the flux distribution to a point solution, or in general, as low-dimensional convex set as possible.

In short, the framework consists of the following steps:

1. The model of the metabolic network of an organism is constructed by selecting a set of biochemical reactions operating in the organism and by designating them to correct cellular compartments;

2. Structural analysis of the isotopomer system is conducted, consisting of the following steps:

(a) Flow analysis of the metabolic network is conducted in order to discover *equivalent fragments*, fragments of carbon backbones of metabolites that will have the same theoretical isotopomer distribution, regardless of the fluxes.

(b) Independence analysis of fragments is conducted to find statistically independent carbon subsets from metabolites, that is, subsets that have been at some point separated along every pathway able to producing them and that have flux invariant isotopomer distributions. This guarantees that the isotopomer distribution of their union assumes the form of a product distribution.

(c) *In silico *calculability analysis is performed to test if the available measurement techniques and substrate labellings make it *in principle *possible to obtain the required flux information.

3. Wet-lab isotopomer tracer experiments are conducted and constraints to isotopomer distributions are measured;

4. The fluxes of the network are estimated. The process consists of the following steps:

(a) Isotopomer measurement data is *propagated *in the metabolic network model from the measured metabolites to unmeasured ones according to the equivalences discovered in step 2.

(b) An equation system tying the isotopomer data and the fluxes together is constructed and solved, either to obtain a flux distribution for the metabolic network as a whole, or for a single junction metabolite to obtain the ratios of fluxes producing it.

(c) The statistical analysis of obtained fluxes or flux ratios is carried out.

In the following we first formalize the ^13^*C *flux analysis problem and then detail the computational steps above.

### Preliminaries

In ^13^*C *metabolic flux analysis the carbon atoms of metabolites are of special interest. We denote with *M *the set of carbon locations *M *= {*c*_1_, ..., *c*_*k*_} of a *k*-carbon metabolite. By |*M*| = *k *we denote the number of carbons in *M. Fragments *of metabolites are subsets *F *= {*f*_1_, ..., *f*_*h*_} ⊆ *M *of the carbons of the metabolite. A fragment *F *of *M *is denoted as *M*|*F*. A *metabolic network G *= (C, R) is composed of a set C = {*M*_1_, ..., *M*_*m*_} of *metabolites *and a set R = {*ρ*_1_, ..., *ρ*_*n*_} of *reactions *that perform the interconversions of metabolites.

With *isotopomers *we mean molecules with similar element structure but different combinations of ^13^*C *labels. Isotopomers of the molecule *M *= {*c*_1_, ..., *c*_*k*_} are represented by binary sequences *b *= {*b*_1_, ..., *b*_*k*_} ∈ {0, 1}^*k *^where *b*_*i *_= 0 denotes a ^12^*C *and *b*_*i *_= 1 denotes a ^13^*C *in location *c*_*i*_. Molecules that belong to the *b-isotopomer *of *M *are denoted by *M*(*b*). Isotopomers of metabolite fragments *M*|*F *are defined in an analogous manner: a molecule belongs to the *F*(*b*)-*isotopomer *of *M*, denoted *M*|*F*(*b*_1_, ..., *b*_*h*_), if it has a ^13^*C *atom in all locations *f*_*j *_that have *b*_*j *_= 1, and ^12^*C *in other locations of *F*. Isotopomers with equal numbers of labels belong to the same *mass isotopomer*. We denote *mass isotopomers *of *M *by *M*(+*p*), where *p *∈ {0, ..., |*M*|} denotes the number of labels in isotopomers belonging to *M*(+*p*).

The *isotopomer distribution D*_*M *_of metabolite *M *gives the relative abundances 0 ≤ *P*_*M*_(*b*) ≤ 1 of each isotopomer *M*(*b*) in the pool of *M *such that

$$\sum_{b\in {\left\{0,1\right\}}^{\left|M\right|}}P_{M}(b)=1.$$

The isotopomer distribution *D*_*M*|*F *_of fragment *M*|*F *and the *mass isotopomer *distribution $D_{M}^{m}$ of mass isotopomers *M*(+*p*) are defined analogously: *D*_*M*|*F *_of metabolite *M *gives the relative abundances 0 ≤ *P*_*M*|*F*_(*b*) ≤ 1 of each isotopomer *M*|*F*(*b*) and $\left(\rho_{1}^{p},\rho_{2}^{p},...,\rho_{n}^{p}\right)$ gives the relative abundances 0 ≤ *P*_*M*_(+*p*) ≤ 1 of each mass isotopomer *M*(+*p*).

**Figure 1:**
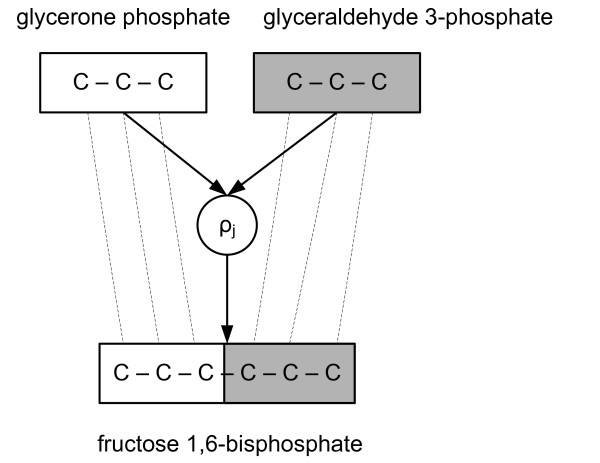
**An example of a metabolic reaction**. In the reaction *ρ*_*j*_, a fructose 1,6-bisphosphate (*C*_6_*H*_14_*O*_12_*P*_2_) molecule is produced from glycerone phosphate (*C*_3_*H*_7_*O*_6_*P*) and glyceraldehyde 3-phosphate (*C*_3_*H*_7_*O*_6_*P*) molecules. Carbon maps are shown with dashed lines. Glyceraldehyde 3-phosphate is equivalent to the gray fragment of fructose 1,6-bisphosphate while glycerone phosphate is equivalent to the white fragment.

Reactions are pairs *ρ*_*j *_= (*α*_*j*_, *λ*_*j*_) where *α*_*j *_= (*α*_1*j*_, ..., *α*_*mj*_) ∈ ℤ^*m *^is a vector of *stoichiometric coefficients*-denoting how many molecules of each kind are consumed and produced in a single reaction event-and *λ*_*j *_is a carbon mapping describing the transition of carbon atoms in *ρ*_*j *_(see Figure 1). Metabolites *M*_*i *_with *α*_*ij *_< 0 are called *substrates *and with *α*_*ij *_> 0 are called *products *of *ρ*_*j*_. If a metabolite is a product of at least two reactions, it is called a *junction*. If *α*_*ij *_< 0, a reaction event of *ρ*_*j *_consumes |*α*_*ij*_| molecules of *M*_*i*_, and if *α*_*ij *_> 0, it produces |*α*_*ij*_| molecules of *M*_*i*_. Bidirectional reactions are modelled as a pair of reactions.

A *pathway p *in network *G *from metabolite fragments {*F*_1_, ..., *F*_*k*_} to fragment *F*' is a sequence $\left(\rho_{1}^{p},\rho_{2}^{p},...,\rho_{n}^{p}\right)$ of reactions such that a *composite carbon mapping *$(\lambda_{n}^{p}\circ \ldots \lambda_{2}^{p}\circ \lambda_{1}^{p})(\cup_{i\in [1,k]}F_{i})=F^{\prime }$, defined by *p *maps the carbons of {*F*_1_, ..., *F*_*p*_} to the carbons of *F*'.

**Figure 2:**
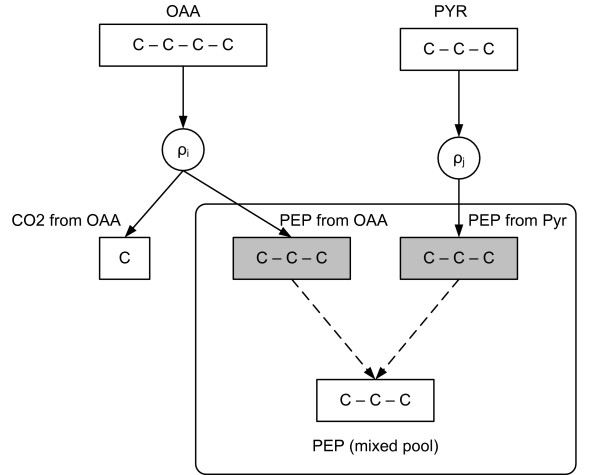
**An example of subpools of a metabolite pool**. Phosphoenolpyruvate (PEP) is produced by two different reactions (*ρ*_*i *_and *ρ*_*j*_), either from Oxaloacetate (OAA) or from glyceraldehyde 3-phosphate (GA3P). Thus, PEP has two in flow subpools, *PEP from OAA *and *PEP from GA3P *(grey boxes) that are mixed in the common PEP pool (at the bottom).

For the rest of the article, it is important to distinguish between the *subpools *of a metabolite pool produced by different reactions. Therefore, we denote by *M*_*ij*_, the subpool of the pool of *M*_*i *_produced (*α*_*ij *_> 0) or consumed (*α*_*ij *_< 0) by reaction *ρ*_*j*_. The concept of the subpools of a metabolite pool is illustrated in Figure 2.

By *M*_*i*0 _we denote the subpool of *M*_*i *_that is related to the external in flow or external out flow of *M*_*i*_. We call the sources of external in flows *external substrates*. Subpools of fragments are defined analogously. In ^13^*C *metabolic flux analysis, the quantities of interest are the rates or the fluxes *v*_*j *_≥ 0 of the reactions *ρ*_*j*_, giving the number of reaction events of *ρ*_*j *_per time unit. We denote by **v **the vector [*v*_1_, ..., *v*_*n*_] of fluxes, or a *flux distribution*.

#### Generalized isotopomer balances

The framework for ^13^*C *metabolic flux analysis presented in this article rests on the assumption that the metabolic network is in metabolic and isotopomeric steady state. In the metabolic steady state, *metabolite balance*, or *mass balance*

(1) $$\sum_{j=1}^{n}\alpha_{ij}v_{j}=\beta_{i}$$

holds for each metabolite *M*_*i*_. Here, *β*_*i *_is the measured external in flow (*β*_*i *_< 0) or external out flow (*β*_*i *_> 0) of metabolite *M*_*i*_. From balance equations (1) defined for every metabolite *M*_*i *_we will obtain a metabolite balancing, or stoichiometric equation system.

In isotopomer steady state, for each isotopomer *b *of each metabolite *M*_*i *_in the metabolic network the following *isotopomer balance *holds:

(2) $$\sum_{j=1}^{n}\alpha_{ij}v_{j}P_{M_{ij}}(b)=\beta_{i}P_{M_{i0}}(b).$$

For metabolic flux analysis (1) and (2) bear a fundamental difference: the former cannot be used to estimate fluxes of alternative pathways producing *M*_*i *_while the latter can. However, using (2) is not in general admissible in practice: typically abundances *P*_*M*_(*b*) of isotopomers are not fully determined from the measurements, and we need to settle for some constraints to the distribution *D*_*M*_. A crucial building block of our framework is the representation of isotopomer measurements as systems of linear equations (c.f. [21])

(3) $$\sum_{b}s_{bh}P_{M}(b)=d_{h},h=1,...,r$$

where *s*_*bh *_is the weight of isotopomer *b *in the *h*'th constraint, *d*_*h *_is a value derived from isotopomer measurements, and *r *is the total number of constraints. We call (3) *isotopomer constraints*. For a *k *carbon metabolite, 2^*k *^linearly independent isotopomer constraints – one for each isotopomer – are necessary and sufficient to constrain the isotopomer distribution *D*_*M *_to a point solution. A set of isotopomer constraints has a natural matrix representation *SD*_*M *_= **d**, where *S *= (*s*_*bh*_)_*b*,*h *_is a 2^*k *^by *r *matrix, where 1 ≤ *r *≤ 2^*k *^is the number of isotopomer constraints (the trivial constraint Σ_*b*_*P*_*M*_(*b*) = 1 by definition always holds).

The use of (3) follows from the simple observation that isotopomer balance (2) implies that each linear combination of isotopomers is balanced. Thus, we can write a new balance equation that constrains the fluxes producing *M*_*i *_as soon as we know the value of the *same *linear combination of isotopomer abundances for each subpool of *M*_*i*_. We have

(4) $$\sum_{j=1}^{n}\alpha_{ij}v_{j}d_{ij}=\beta_{i}d_{i0},$$

where each $d_{ij}=\sum_{b}s_{b}P_{M_{ij}}(b)$ is a linear combination of the form (3), with coefficients *s*_*b *_that do not depend on *j*, i.e. they are the same for each subpool *M*_*ij*_. We call (4) a *generalized isotopomer balance*.

#### Representing MS and NMR isotopomer measurements as linear constraints

In the following, we will show by examples how MS and NMR data can be represented as isotopomer constraints. Let us first consider mass isotopomer distributions obtained from MS. Here we omit discussion on practicalities such as corrections for natural abundances of ^13^*C *isotopes (c.f., [6,36]) and concentrate on the conceptual level. For example, the +2 mass isotopomer of a three carbon metabolite *M *satisfies

*P*_*M*_(+2) = *P*_*M*_(011) + *P*_*M*_(110) + *P*_*M*_(101)

which conforms to (3) by taking *s*_011,2 _= *s*_110,2 _= *s*_101,2 _= 1, and *s*_*b*,2 _= 0 otherwise. Similarly, the coefficients *s*_*bk *_can be derived for all mass isotopomers +*k*, *k *= 0, ..., 3.

Isotopomer data originating from Tandem MS, or MS-MS, falls into the same representation. Consider, for example, a fragment *M*|*F *of a three-carbon metabolite, containing the first and second carbon of *M*. The following equation holds for the mass isotopomer *M*|*F*(+1):

*P*_*M*|*F*_(+1) = *P*_*M*|*F*_(01) + *P*_*M*|*F*_(10).

The equation can be written in terms of the precursor *M*, but the exact form of the equation depends on the mode of tandem MS. If the full scan mode is used, we obtain

*P*_*M*|*F*_(+1) = *P*_*M*_(010) + *P*_*M*_(011) + *P*_*M*_(100) + *P*_*M*_(101),

as all precursor molecules *M *that have exactly one carbon among the first and second location contribute to the fragment mass isotopomer *M*|*F*(+1). On the other hand, in the daughter-ion scanning mode a single mass isotopomer, for example *M*(+2), is selected as the precursor. Then we obtain

*P*_*M*|*F*_(+1) = *P*_*M*_(011) + *P*_*M*_(101),

as the precursor must always have two ^13^*C *atoms in total. We refer the reader to [6,36] for further details. Also NMR ^13^*C *isotopomer measurements, where relative intensities of different combinations of ^13^*C *and ^12^*C *atoms that are coupled to an observed ^13^*C *atom are measured, can be expressed as linear combinations of isotopomer abundances. For example, for a three-carbon metabolite *M*, the following constraints to $D_{M_{i}}$ can be inferred for the labeling pattern 010:

$$\frac{P_{M}(010)}{\sum_{b_{1},b_{3}\in \{0,1\}}P_{M}(b_{1}1b_{3})}=d(010)$$

where *d*(010) is the measured intensity. Rewriting the above as

$$d(010)\cdot \sum_{b\in \{110,011,111\}}P_{M}(b)=(1-d(010))\cdot P_{M}(010)$$

and denoting *s*_*b *_= *d*(010), for *b *∈ {110, 011, 111}, *s*_010 _= *d*(010) - 1 and *s*_*b *_= 0 for *b *∈ {000, 100, 001, 101}, the above can be seen to conform to (3). Similar derivation can be used for other isotopomer signals present in the NMR spectrum to obtain the corresponding isotopomer constraints.

#### Projection of isotopomer measurements to fragments

In our computational framework, it will be necessary to project the measurement data obtained for a metabolite *M *to its fragments *M*|*F *and vice versa. In this projection, we want to avoid any unnecessary loss of measurement information, that is, we want to obtain as many linearly independent constraints to the isotopomer distribution of *F *as possible. For example, if we have measured that a two-carbon metabolite *M *has the isotopomer distribution

*P*_*M*_(00) = 0.2, *P*_*M*_(01) = 0.3, *P*_*M*_(10) = 0.4, *P*_*M*_(11) = 0.1

and we need to know the isotopomer distribution of the fragment *M*|*F *consisting of the first carbon of *M*, we should obtain

$$\begin{array}{c} P_{F}(0)=P_{M}(0*)=P_{M}(00)+P_{M}(01)=0.5,\\ P_{F}(1)=P_{M}(1*)=P_{M}(10)+P_{M}(11)=0.5. \end{array}$$

For the general model of isotopomer measurements (4) the projection of measurement information from a metabolite to its fragments can be done by the techniques of linear algebra introduced in [21]. We recapitulate the techniques in the following.

Recall the general form of isotopomer measurement *SD*_*M *_= **d**, where *S *denotes a matrix with 2^*k *^columns, one column for each isotopomer *b *of *k*-carbon metabolite *M*, and each row *h *of *S *corresponds to a measured isotopomer constraint (3). The rows of *S *span a subspace $S\subseteq I_{M}$ in a 2^*k *^dimensional vector space $I_{M}$ spanned by all possible isotopomer distributions *D*_*M*_.

Also the metabolite fragments are naturally represented as vector subspaces. Let *U*_*F *_denote a matrix with also a column for each isotopomer *M*(*b*) and a row for each isotopomer *F*(*b*') of *M*|*F*, that is,

(5) $$U_{F}(b^{\prime },b)=\{\begin{array}{ll} 1 & \begin{array}{cc} \text{if }b_{j}={b^{\prime }}_{j} & \text{for all carbon positions }j\in F_{k} \end{array}\\ 0 & \text{otherwise}. \end{array}$$

The rows of *U*_*F *_span another subspace $U_{F}\subseteq I_{M}$. Any isotopomer distribution *D*_*M*|*F *_lies in this subspace, and hence also any isotopomer constraint *S*_*F*_*D*_*M*|*F *_= **d **for fragment *M*|*F *necessarily lies in the same subspace.

In conclusion, the available information about *D*_*M *_is given as its linear projection onto S, and anything we can express about *D*_*M*|*F *_in terms of isotopomer constraints is contained within $U_{F}$. Thus, any isotopomer constraint for *D*_*M*|*F *_that we can derive from the measurements can be expressed in terms of the *vector space intersection *$S\cap U_{F}$.

Thus, to obtain isotopomer constraints for fragment *M*|*F *from a measurement *SD*_*M *_= **d**, we need to compute the vector space intersection $Y_{M|F}=S\cap U_{F}$ and project the measurement to $Y_{i,F}$. This can be done by standard linear algebra (c.f. [21]). This process gives us as output isotopomer constraints of the required form

*Y*_*F*_*D*_*M*|*F *_= **d**_*F*_.

Finally, transforming a fragment constraint *Y*_*F*_*D*_*M*|*F *_= **d**_*F *_into an isotopomer constraint *SD*_*M *_= **d **is easy: we postmultiply the fragment constraint with the matrix *U*_*M*|*F *_: *S *= *Y*_*F*_*U*_*M*|*F *_and **d **= **d**_*F*_*U*_*M*|*F*_.

### Structural analysis of isotopomer systems

The incomplete nature of ^13^*C *measurement data requires us to find ways to use the available data the best way possible. The central concept is to find invariants of isotopomer distributions that remain through the pathways, and allow us to trade or *propagate *measurement information from one metabolite to another. This allows us to write or augment generalized isotopomer balances for metabolites for which the isotopomer distributions are not completely determined by measurements. Thus, the fluxes are potentially more tightly pinpointed as well.

In particular, we use two techniques: First, *flow analysis *is used to uncover sets of metabolite fragments that have the same isotopomer distribution regardless of the fluxes. Second, *independence analysis *of fragments is used to uncover situations where two fragments of the same metabolite induce the product distribution for the isotopomer distribution of their union.

#### Flow analysis of metabolic networks

The goal of the flow analysis [27] is to partition the fragments of the metabolites in the network to *equivalence classes *such that fragments in the same equivalence class have identical isotopomer distributions in every steady state. This can be guaranteed if a fragment is produced from another a such a manner that the carbons within the fragment never depart from each other regardless of the pathway that is being used.

Formally, we say that fragment *F*' *dominates *fragment *F *if the following conditions are met

1. *F *and *F*' have the equal number of carbons;

2. all carbons of *F *originate always from the carbons of *F*';

3. carbon of *F*' stay connected to each other via all pathways from *F*' to *F*;

4. composite carbon mappings are the same in all pathways from *F*' to *F*.

Intuitively, a *dominated fragment *(*F*) is always produced from its *dominator *(*F*') without manipulating the carbon chain of the fragment. Thus, isotopomer distribution of the dominated fragment does not contain any information about the metabolic fluxes. For a fragment *F *that has no dominators, the transitive closure of the domination relation corresponds to the class of equivalent fragments in the network.

The simplest example of fragment equivalence is the one between a substrate *M*_*k *_and product *M*_*i *_in a single reaction *ρ*. If the atoms in *M*_*i*_|*F *originate from *M*_*k*_|*F*', then the fragments *M*_*k*_|*F*' in the subpool *M*_*ij *_produced by reaction *ρ*_*j*_, are equivalent with the fragment *M*_*i*_|*F *(Figure 1). Furthermore, if metabolite *M*_*i *_has only one producing reaction *ρ*_*j*_, isotopomer distributions of subpool *M*_*ij *_and *M*_*i *_coincide. Thus, if fragment *M*_*i*_|*F *is produced from a single fragment *M*_*k*_|*F*' of some substrate *M*_*k *_of *ρ*_*j*_, *F *and *F*'are equivalent. By transitivity, all fragments in the linear pathway are equivalent.

**Figure 3:**
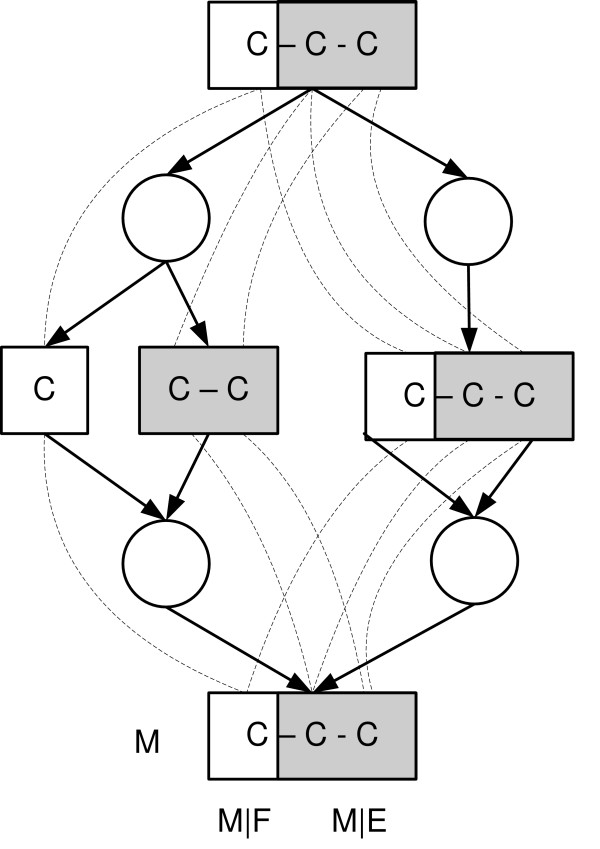
**An example of fragment equivalence classes in a branched pathway**. An example of equivalence classes of fragments in the metabolic network that contains dominated junction fragments *M*|*E *and *M*|*F*. Grey and white fragments constitute two equivalence classes. Dashed lines depict carbon mappings.

**Figure 4:**
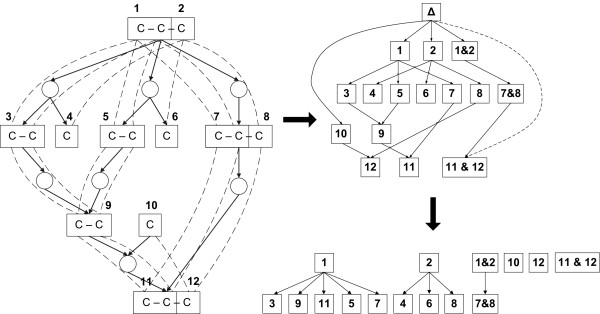
**An example of a fragment flow graph and a dominator tree**. A metabolic network (left), the corresponding fragment flow graph (up right) and the subtrees of the dominator tree (down right).

More complicated case of fragment equivalence is found when a fragment of a junction metabolite is dominated by an upstream fragment (Figure 3). In [27] we show that the the classes of equivalent fragments corresponding the conditions (1–4) can be efficiently computed. Very brie fly, first the metabolic network is transformed to a *fragment flow graph *that connects substrate metabolite fragments to their product fragments for each reaction in the network. Then, the dominator tree [37,38] of the fragments in the fragment flow graph is constructed. It turns out that the subtrees of this dominator tree correspond to the required fragment equivalence classes (see Figure 4).

Fragment equivalence classes have many uses [27]. Most importantly, measured isotopomer constraints to fragment *F *can be directly propagated to another fragment *F' *in the same equivalence class, by applying the joint carbon mappings between *F *and *F*'. This helps in the construction of generalized balance equations (4) where the same isotopomer information is required for each subpool of junction metabolites.

#### Independence analysis of fragments

A complementary property to fragment equivalence ^13^*C *is the statistical independence of fragment isotopomer distributions. Intuitively, if two fragments of the same metabolite are statistically independent, new isotopomer constraints to the union of them can be obtained by taking a product of the isotopomer distributions of the independent fragments.

More formally, the basic question is on what conditions the distribution *D*_*M*|*E*∪*F *_of union of two fragments will necessarily have the form of a *product distribution*

(6) *D*_*M*|*E*∪*F *_= *D*_*M*|*E *_⊗ *D*_*M*|*F*_

where ⊗ denotes the tensor product consisting all terms of the form *P*_*E*∪*F*_(*b*) = *P*_*E*_(*b*')·*P*_*F*_(*b*"), where *b*' (resp. *b*") ranges over all isotopomers of *E *(resp. *F*), and *b *is the isotopomer of *M*|*E *∪ *F *formed by joining *E *and *F*.

The utility of fragment independence is in that it gives us constraints to the isotopomer distributions that are complementary to the isotopomer constraints (3) obtained from the measurements.

In general, two criteria need to be satisfied for statistical independence of two fragments *M*|*E *and *M*|*F*. First, the fragments need to be *structurally *independent, meaning that along all pathways producing the metabolite, at some point all carbons of the fragments have resided in different metabolite molecules. This property can be defined in recursive manner. Fragments *M*|*E *and *M*|*F *are structurally independent if for all carbon pairs (*a*, *b*), *a *∈ *E *and *b *∈ *F*, for all reactions *ρ *producing *M*, it holds that

• *a *and *b *originate from different reactants of *ρ*, or

• *a *and *b *originate from the same reactant *M*', and the reactant fragments *M*'|*F*_*a *_and *M*'|*F*_*b*_, where *F*_*a *_= $\lambda_{\rho }^{-1}$(*a*), *F*_*b *_= $\lambda_{\rho }^{-1}$(*b*), are structurally independent.

The second necessary condition is that the two fragments need to be dominated by some other metabolite fragments in the network. This will make the fragment distributions flux invariant. Together, the two criteria guarantee (6) to hold.

A simple case of statistical independence of fragments is a (subpool) product metabolite *M*_*i *_of a single reaction *ρ*_*j*_, where the fragments *M*_*i*_|*E *and *M*_*i*_|*F *are disjoint and originate from different reactants. The fragments are structurally independent (by originating from different reactants) and are dominated (by reactant fragments of *ρ*_*j*_). Hence (6) holds. The underlying assumption here is that enzymes pick their reactants independently and randomly from the available pools. This case of statistical independence of fragments is depicted Figure 1, where white and grey fragments of D-fructose 1,6-biphosphate are statistically independent (in the subpool of reaction *ρ*_*j*_).

**Figure 5:**
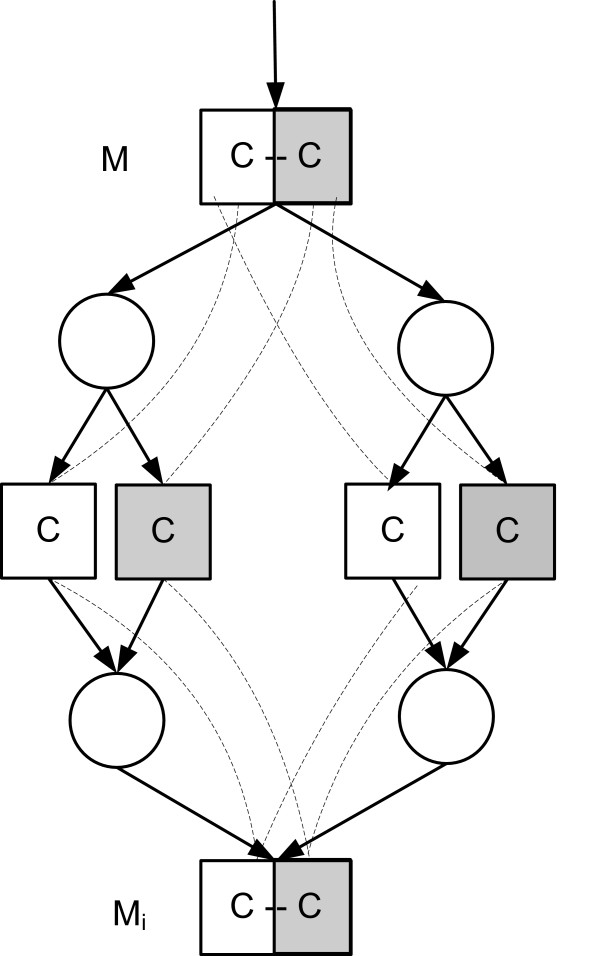
**An example of statistical independence of fragments**. White and grey one-carbon-fragments of *M*_*i *_are statistically independent: both fragments are dominated by one-carbon-fragments of *M*, and the fragments are disjoint in every pathway that produce *M*_*i *_from *M*.

Another simple example is a junction metabolite *M*_*i *_that has two or more producers with associated subpools *M*_*ij*_. If *M*_*ij*_|*E *and *M*_*ij*_|*F *are structurally independent in all subpools, *M*_*i*_|*E *and *M*_*i*_|*F *are structurally independent as well. If *M*_*i*_|*E *and *M*_*i*_|*F *are dominated by some fragments in the network, all subpools have the same distribution which takes the form of (6). Without dominance the distribution $D_{M_{i}|E\cup F}$ will in general be a *flux-dependent mixture *of product distributions $D_{M_{i}|E\cup F}=\sum_{j}v_{j}D_{M_{ij}|E}⊗D_{M_{ij}|F}$. This case of statistical independence is depicted in Figure 5.

A generalized form of (6), useful for propagation of isotopomer constraints, is derived as follows. Assume independent fragments *M*|*E *and *M*|*F *of metabolite *M *and isotopomer constraints *S*_*E*∪*F*_*D*_*M*|*E*∪*F *_= **d**_**E∪F**_, *S*_*E*_*D*_*M*|*E *_= **d**_**E **_and *S*_*F*_*D*_*M*|*F *_= **d**_*F*_, where *S *= *S*_*E *_⊗ *S*_*F*_. Now, the the *h*'th constraint for fragment *E *and *g*'th constraint for fragment *F*, written as Σ_*a*_*s*_*ah*,*E*_*P*_*M*|*E*_(*a*) = *d*_*h*,*E *_and Σ_*c*_*s*_*cg*,*F*_*P*_*M*|*F*_(*c*) = *d*_*g*,*F*_.

Multiplying the constraints, and denoting *s*_*b *_= *s*_*ah*,*E*_·*s*_*cg*,*F *_where *b *is the isotopomer consistent with fragment isotopomers *a *and *c*, we get the following equation

$$d_{h,E}\cdot d_{g,F}=\left(\sum_{a}s_{ah,E}P_{M|E}(a)\right)\cdot \left(\sum_{a}s_{cg,F}P_{M|F}(a)\right)=\sum_{a}s_{b}P_{M|E\cup F}(b)=d_{l,E\cup F},$$

for the *l*'th constraint for *E *∪ *F*. The equations of the above kind can be concisely written in terms of tensors:

(7) **d **= *SD*_*M*|*E*∪*F *_= (*S*_*E *_⊗ *S*_*F*_) *D*_*M*|*E *_⊗ *D*_*M*|*F *_= **d**_*E *_⊗ **d**_*F*_.

From above, if two of the three vectors **d**, **d**_*E*_, **d**_*F *_are known, the remaining unknown one can be solved.

We note in passing that computing constraints to the metabolite given constraints to the fragments is straightforward application of (7).

#### Applying fragment independence analysis to flux ratio computation

In our framework, statistical independence of fragments has two uses. We apply it

1. to compute isotopomer constraints for the union of independent fragments, given isotopomer constraints to its independent fragments, and

2. to compute isotopomer constraints for an independent fragment given isotopomer constraints to the other fragments and the metabolite as a whole.

In both cases making use of (6) gives us a larger set of constraints than the vector space and flow analysis approach alone.

**Figure 6:**
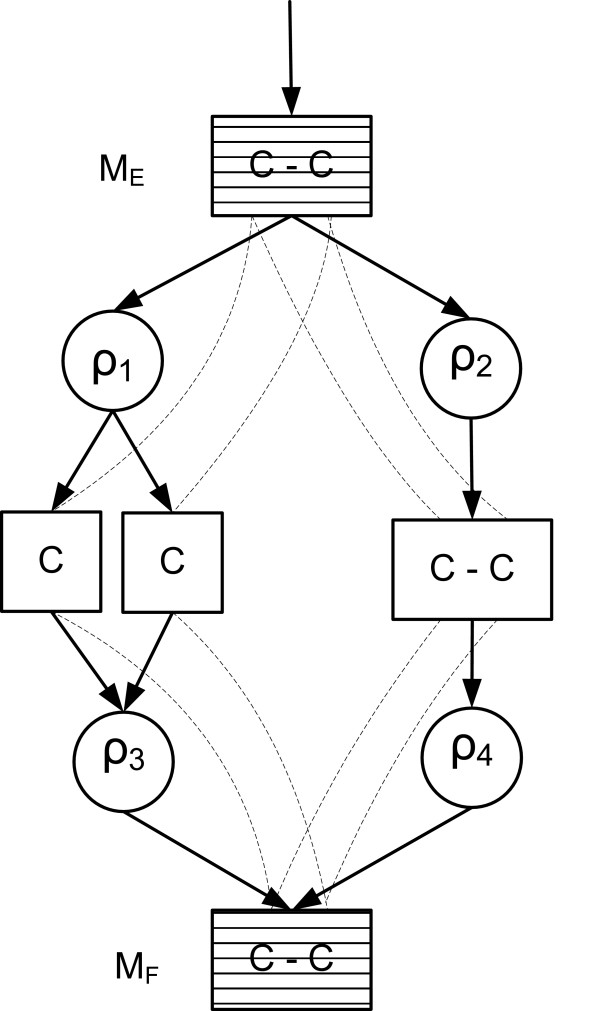
**An example of using fragment independence to obtain new isotopomer constraints under uniform substrate labelling**. Constraints to the isotopomer distributions of striped metabolites are assumed to be known, either by direct measurement of measurement propagation. In pathway *q *= (*ρ*_2_, *ρ*_4_), the isotopomer distribution of *M*_*F *_molecules will be the same as in *M*_*E*_. In pathway *p *= (*ρ*_1_, *ρ*_3_), the isotopomer distribution of *M*_*F *_can be derived by applying fragment independence: the isotopomer distributions of single carbon metabolites produced by *ρ*_1 _are known *a priori *to be equal to the labelling degree of uniformly labelled substrate. As the two carbons of *M*_*F*3 _are produced from two different metabolites, these carbons are statistically independent to each other in the subpool and the isotopomer distribution *D*($M_{F_{p}}$) of *M*_*F *_molecules produced by *p *can be computed by applying Equation 7.

Next we describe how (7) generalizes the basic measurement propagation step of the traditional metabolic flux ratio analysis [31]. In the basic case, the flux ratios are solved for two competing pathways *p *and *q*, which *p *cleaves a certain carbon-carbon bond *b *of junction *M *while the *q *preserves *b *intact from the external substrate. (See Figure 6 for an example). This serves also as an example of applying (7) to compute isotopomer constraints for the union of independent fragments.

When a uniformly labelled substrate is used, the labelling degree of every carbon in the network is the same (and known *a priori*) when the system reaches isotopomeric steady state. Thus, the isotopomer distribution $D_{F_{p}}$ of a two-carbon fragment *F *(metabolite *M*_*F *_in Figure 6) containing bond *b *can be computed by (7) for pathway *p *cleaving *b*, while for pathway *q*, $D_{F_{q}}$ can be propagated from the external substrate (metabolite *M*_*E *_in Figure 6) using the fragment equivalence classes of the previous section. If we are able to measure (constraints to) the isotopomer distribution of the mixed pool *F*, we can then automatically derive a generalized isotopomer balance corresponding the manually derived ratio. To use (7) to compute isotopomer constraints for an independent fragment from the known isotopomer constraints to the other independent fragment and to the whole metabolite is complicated by the incompleteness of the measurement data: an arbitrary measurement *SD*_*M*|*E*∪*F *_= **d **might not be directly representable via a tensor product *S *= *S*_*E *_⊗ *S*_*F*_. Instead, we need to first compute isotopomer subspaces for known isotopomer constraints where (7) can be applied.

**Figure 7:**
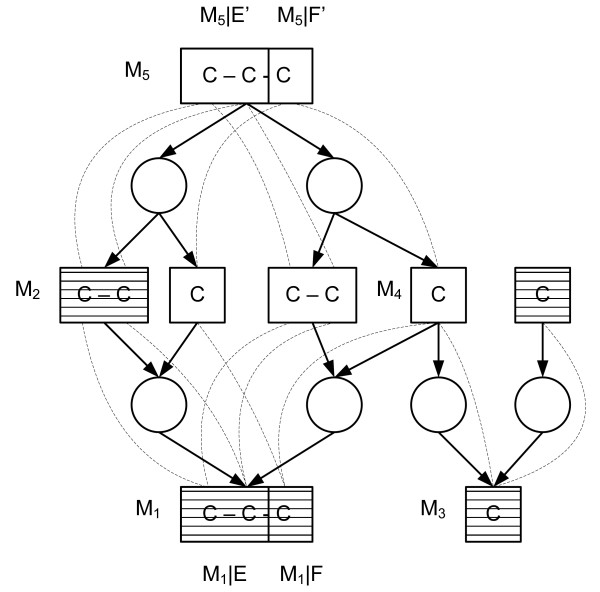
**An example of using fragment independence to obtain new isotopomer constraints for a reactant**. The mass isotopomer distributions of striped metabolites are assumed to be measured. Fragments *M*_1_|*E *and *M*_2 _belong to the same fragment equivalence class. Thus, *D*_*m*_(*M*_1_|*E*) can be derived from *D*_*m*_(*M*_2_) by the measurement propagation inside equivalence classes. Furthermore, fragments *M*_5_|*E*' and *M*_5_|*F*' dominate fragments *M*_1_|*E *and *M*_1_|*F*, and the bond between *M*_1_|*E *and *M*_1_|*F *is broken in all pathways producing *M*_1 _from *M*_5_. Thus, *M*_1_|*E *and *M*_1_|*F *are statistically independent, and *D*_*m*_(*M*_1_|*F*) can be deduced from *D*_*m*_(*M*_1_) and *D*_*m*_(*M*_1_|*E*) by utilizing Equation 7. Computed *D*_*m*_(*M*_1_|*F*) can then be propagated to *M*_4_, as *M*_1 _and *M*_4 _belong to the same fragment equivalence class. Finally, *D*_*m*_(*M*_4_) helps to solve the ratios of fluxes entering to *M*_3_.

The detailed description of this technique is rather technical and omitted from this article. Here we give an example of the technique (See Figure 7). We assume that we know the mass isotopomer distributions $D_{M_{i}}^{m}$ of metabolite *M*_*i *_(metabolite *M*_1 _in Figure 7) and $D_{M_{i}|E}^{m}$ of fragment *M*_*i*_|*E*. We furthermore assume that *M*_*i*_|*E *and *M*_*i*_|*F *are independent. From this information the mass isotopomer distribution of $D_{M_{i}|E}^{m}$ can be solved. To be exact, $D_{M_{i}|E}^{m}$ can be solved from the system containing an equation

(8) $$P_{M_{i}}(+p)=\sum_{k+l=p}P_{E}(+k)\cdot P_{F}(+l)$$

for each mass isotopomer *p *of *M*_*i*_. To see that (8) conforms to (7), we denote the measured mass isotopomer distribution $D_{M_{i}}^{m}$ = *S*·*D*(*M*_*i*_) of *M*_*i *_by **d**_*M *_(i.e. rows of coefficient matrix *S *correspond to different mass isotopomers of *M*_*i*_) and the measured mass isotopomer distributions of *E *and *F *by **d**_*E *_and **d**_*F*_. Let |*E*| = 2, |*F*| = 1 and |*M*_*i*_| = 3, thus *M*_*i *_= *E *∪ *F*. We have

$$S_{E}=\left(\begin{array}{cccc} 1 & 0 & 0 & 0\\ 0 & 1 & 1 & 0\\ 0 & 0 & 0 & 1 \end{array}\right),S_{F}=\left(\begin{array}{cc} 1 & 0\\ 0 & 1 \end{array}\right).$$

with the tensor product

$$S_{E}⊗S_{F}=\left(\begin{array}{cccccccc} 1 & 0 & 0 & 0 & 0 & 0 & 0 & 0\\ 0 & 1 & 0 & 0 & 0 & 0 & 0 & 0\\ 0 & 0 & 1 & 0 & 1 & 0 & 0 & 0\\ 0 & 0 & 0 & 1 & 0 & 1 & 0 & 0\\ 0 & 0 & 0 & 0 & 0 & 0 & 1 & 0\\ 0 & 0 & 0 & 0 & 0 & 0 & 0 & 1 \end{array}\right),\text{and }S=\left(\begin{array}{cccccccc} 1 & 0 & 0 & 0 & 0 & 0 & 0 & 0\\ 0 & 1 & 1 & 0 & 1 & 0 & 0 & 0\\ 0 & 0 & 0 & 1 & 0 & 1 & 1 & 0\\ 0 & 0 & 0 & 0 & 0 & 0 & 0 & 1 \end{array}\right).$$

As the two matrices are not the same (7) is not directly applicable. However, by summing up the second and the third rows and the fourth and the fifth rows of *S*_*E *_⊗ *S*_*F *_we obtain *S*. Intuitively, this means that Equation (7) can be applied to compute $D_{M|F}^{m}$, when we take into account that the isotopomer constraints corresponding both second and third rows of *S*_*E *_⊗ *S*_*F *_contribute to mass isotopomer *M*_*i*_(+1), while the isotopomer constraints corresponding fourth and fifth rows of *S*_*E *_⊗ *S*_*F *_contribute to mass isotopomer *M*_*i*_(+2). (From the definition of the tensor product we see that, for example, the second row of *S*_*E *_⊗ *S*_*F *_corresponds to isotopomer constraints *P*_*E*_(00)·*P*_*F*_(1) = *P*_*E*_(+0)·*P*_*F*_(+1) and the third row corresponds to the constraints *P*_*E*_(01)·*P*_*F*_(0) + *P*_*E*_(10)·*P*_*F*_(0) = *P*_*E*_(+1)·*P*_*F*_(+0), thus validating our intuitive observation.) When the similiar information for all rows of *S*_*E *_⊗ *S*_*F *_is collected to a linear equation system, we will obtain the following constraints to the mass isotopomer distribution $D_{M|F}^{m}$ (which in the case of one-carbon-fragment *M*|*F *coincides with the isotopomer distribution *D*_*M*|*F*_):

$$\left(\begin{array}{cc} P_{E}(+0) & 0\\ P_{E}(+1) & P_{E}(+0)\\ P_{E}(+2) & P_{E}(+1)\\ 0 & P_{E}(+2) \end{array}\right)D_{m}(M|F)=d_{M}=\left(\begin{array}{c} P_{M_{i}}(+0)\\ P_{M_{i}}(+1)\\ P_{M_{i}}(+2)\\ P_{M_{i}}(+3) \end{array}\right)=SD_{M_{i}},$$

which is equal to (8).

#### Calculability analysis

Isotopomer tracer experiments using less common carbon sources can be very costly because of the prices of purposefully labelled substrates. Thus, it is very useful to be able to first conduct *in silico *calculability analysis to find out, whether it is even in principle possible to obtain the required flux information from the tracer experiment. By analyzing the fragment equivalence classes, it is relatively easy to perform this kind of "structural identifiability analysis" (cf. [39,40] for global isotopomer balancing), that is, to discover the set of junction metabolites for which the flux ratios can be calculated (in the best case) from the given measurement data: it is enough to check what type of isotopomer constraints

(9) $$\sum_{b}s_{bij}P_{M_{ij}}(b)=d_{ij},$$

can be propagated to each subpool *M*_*ij *_of junction metabolites *M*_*i *_from the measured metabolites (we need to know only coefficients *s*_*bij*_, not the isotopomer abundances *d*_*ij*_). Then, by applying the techniques of computing vector subspace intersection described above, we can compute the maximal number of linearly independent constraints obtainable for the flux ratios of each junction. Thus, it is possible to check before costly and time-consuming wet lab experiments, whether the experiments even have potential to answer the biological questions at hand. The results of the calculability analysis tell which flux ratios are in principle determinable, given the labelling of external substrates, topology of the metabolic network and the available measurement data. It then depends on the actual flux distribution and the accuracy of the measurements, whether these ratios can be reliably determined from the experimental data.

### Estimating the flux distribution of the metabolic network

In the main step of our framework for ^13^*C *metabolic flux analysis, the fluxes of the metabolic network are estimated by forming and solving generalized isotopomer balance equations (4). The generalized isotopomer balance equations are based on the isotopomer measurement data that is first propagated in the network to unmeasured metabolites by utilizing the results of the structural analysis presented above.

#### Measurement propagation

The aim of the propagation of measurement data is to infer from the isotopomer constraints of measured metabolites as many isotopomer constraints as possible to unmeasured metabolites. As a rule of thumb, more constraints the unmeasured metabolites will get more generalized isotopomer balance equations (4) bounding the fluxes can be written.

Fragment equivalence classes can be utilized in the measurement propagation: from isotopomer constraints known for fragment *M*_*i*_|*F *isotopomer constraints for other fragments *M*_*l*_|*F*_*k *_in the equivalence class of *F *can be easily computed. The process is the following:

1. Before measurements are propagated from fragment *M*|*F *of measured metabolite *M *to other fragments in the equivalence class of *F*, isotopomer constraints to *F *are computed from the constraints measured to the whole metabolite *M *by using the vector space projection techniques (see Section *Projection of isotopomer measurements to fragments*).

2. The fragment constraints are propagated to all fragments *F*' that have been found equivalent to *F *via the flow analysis technique.

This requires mapping of isotopomers of *F *to isotopomers of *F*' by applying the carbon mappings of the reactions along any pathway between *F *and *F*'.

3. After the propagation of measurement data inside the fragment equivalence classes, new isotopomer constraints for independent fragments of the same metabolite can be derived, as described in Section *Independence analysis of fragments*.

Steps 2 and 3 can be iterated until no new isotopomer constraints to the fragments are discovered.

#### Construction of generalized isotopomer balances

After the propagation step, we have some isotopomer constraints $S_{ij}D_{M_{ij}}=d_{ij}$ for each subpool *j *of every junction metabolite *M*_*i*_. (For non-junction metabolites, isotopomer balance equations do not contain any additional flux information compared to the mass balances.) In the best case we know complete isotopomer distribution $D_{M_{ij}}$, in the worst case we have only trivial constraints stating that the sum of relative abundances of all isotopomers equals one.

Next, a linear equation system containing flux constraints obtained from mass balances (1) and generalized isotopomer balances (4) is constructed.

However, the isotopomer constraints of different subpools do not yet conform to (4) as the matrices *S*_*ij *_are not necessarily the same.

Thus we still need to compute a common subspace $Y_{i}=\cap_{j}S_{ij}$ ($S_{ij}$ is spanned by the rows of *S*_*ij*_) of the isotopomer constraints known for each subpool *M*_*ij *_and project subpool constraints $S_{ij}D_{M_{ij}}=d_{ij}$ to $Y_{i}$.

**Figure 8:**
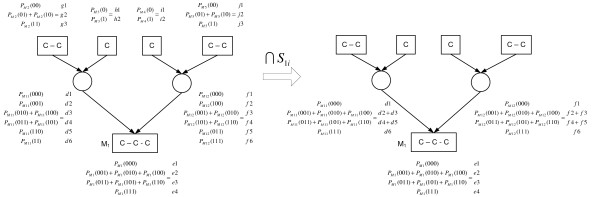
**An example of the computation of the common subspace of isotopomer constraints in different subpools**. The mass isotopomer distribution of junction metabolite *M*_1 _is assumed to be measured. For the in flow subpools *M*_11 _and *M*_12 _we obtain isotopomer constraints from the above reactant metabolites by measurement propagation. These propagated constraints must be projected to mass isotopomer to the subspace defined by the mass isotopomer distribution of *M*_1 _before generalized isotopomer balances are constructed.

This can be done with the same techniques that were previously applied to project measured isotopomer information of a metabolite to its fragments. Let *Y*_*i *_be a matrix with row space $Y_{i}$. After the projection we obtain isotopomer constraints $Y_{i}D_{M_{ij}}=z_{ij}$ for each subpool *M*_*ij *_(See Figure 8 for an example).

Now the isotopomer constraints of all the subpools lie in the same subspace of $I_{M_{i}}$ and we are ready to write the system of generalized isotopomer balance equations (4) for every junction *M*_*i*_:

(10) $$\sum_{j=1}^{n}\alpha_{ij}v_{j}z_{ij}=\beta_{i}z_{i0},$$

that is,

(11) $$A_{i}v=\left[\begin{array}{ccc} \alpha_{1i}{\left(z_{1i}\right)}_{1} & \cdots & \alpha_{ni}{\left(z_{ni}\right)}_{1}\\ \vdots & \ddots & \vdots \\ \alpha_{1i}{\left(z_{1i}\right)}_{r} & \cdots & \alpha_{ni}{\left(z_{ni}\right)}_{r} \end{array}\right]\cdot \left[\begin{array}{c} v_{1}\\ \vdots \\ v_{n} \end{array}\right]=g_{i},$$

where **g**_*i *_= *β*_*i*_**z**_*i*0_.

#### Estimating the fluxes

The ratios of (forward) fluxes producing *M*_*i *_can be computed by solving the corresponding Equation (11) augmented with a constraint that fixes the out flow from *M*_*i *_to equal 1. Thus, we obtain flux ratios of junction metabolites without manual derivation of ratio equations, without nonlinear optimization and without knowing intake and outtake rates of external metabolites or biomass composition.

In addition, when the equations (11) of all junction metabolites are combined with the mass balances (1) of non-junctions, we obtain a linear equation system

(12) $$Av=\left(\begin{array}{c} A_{1}\\ \vdots \\ A_{m} \end{array}\right)\cdot \left[\begin{array}{c} v_{1}\\ \vdots \\ v_{n} \end{array}\right]=\left[\begin{array}{c} g_{1}\\ \vdots \\ g_{m} \end{array}\right]=g$$

constraining the fluxes **v **of the network that contains a block (junctions) or a row (non-junctions) *A*_*k *_for each metabolite *M*_*k*_. Measured external fluxes and other known constraints, such as the composition of biomass can also be added to (12) as additional constraints. Additional constraints, like ones derived from gene regulatory information [41] or from thermodynamic analysis of metabolism [42-44] can also easily be included to (12).

If (12) is of full rank, the whole flux distribution can be solved with standard linear algebra [45]. Also, more complex, nonlinear methods can be applied to model the effect of experimental errors to the estimated flux distribution [20]. In a common case where the system is of less than full rank, a single flux distribution can not be pinpointed without additional constraints. Instead, (12) defines the space of feasible flux distributions, that are all equally good solutions. In that case we can apply techniques developed for the analysis of stoichiometric matrices to determine as many fluxes as possible [46] from (12). More generally, by linear programming we can obtain maximum (resp. minimum) values for each flux *v*_*i*_:

(13) $$\begin{array}{l} \text{For each }v_{i}:\\ \begin{array}{cc} \max & v_{i} \end{array}\\ \begin{array}{cc} \text{s}\text{.t}\text{.} & Av=g \end{array}\\ v_{i}^{min}\leq v_{i}\leq v_{i}^{max}\forall v_{i}\in v, \end{array}$$

where $v_{i}^{min}$ and $v_{i}^{max}$ are predetermined minimum and maximum allowable values for *v*_*i*_

Furthermore, it is possible to search for in some sense optimal flux distribution – for example a flux distribution maximizing the production of biomass – from the feasible space defined by (12) by linear programming techniques of flux balance analysis [1,3,47,48]. In that case, isotopomer data constrain the feasible space more than the stoichiometric information would alone do, thus possibly allowing more accurate estimations of the real flux distribution.

### Statistical analysis

For an experimentalist, it is important to know how sensitive the obtained estimation of fluxes is to measurement errors. If enough repeated measurements are not available to assess this sensitivity, it has to be estimated by computational techniques. In the global isotopomer balancing framework for ^13^*C *metabolic flux analysis, many mathematically or computationally involved methods have been developed to analyze the sensitivity of estimated flux distributions to errors in isotopomer measurements and the sensitivity of the objective function to the changes in the generated candidate flux distributions [49-53].

As our direct method for ^13^*C *metabolic flux analysis is computationally efficient, we can afford to a simple, yet powerful Monte Carlo procedure to obtain estimates on the variability of individual fluxes due to measurement errors:

1. For each measured metabolite *M*_*i*_: By studying the variability in the repeated measurements, fix the distribution *Ω*_*i *_from which the measurements of *M*_*i *_are sampled.

2. Repeat *k *times:

(a) For each measured metabolite *M*_*i*_: sample a measurement from *Ω*_*i*_.

(b) Estimate fluxes **v**_*l *_from the sampled measurements.

3. Compute appropriate statistics from the set *V *= {**v**_1_, ..., **v**_*k*_} to describe the sensitivity of fluxes to measurement errors.

Possible statistics that can be applied in the last step of the above algorithm include standard deviation, empirical confidence intervals [53], kurtosis, standard error etc. of each individual flux *v*_*j *_and measures of "compactness" of *V*, such as (normalized) average distance of items of *V *from the sample average.

### Experimental NMR and GC-MS methods

In this section we shortly describe the experimental procedures applied in NMR and GC-MS isotopomer measurements that produced the data for Section.

In the first experiment *S. cerevisiae *was grown in an aerobic glucose-limited chemostat culture at dilution rate 0.1 *h*^-1^. After reaching a metabolic steady state, as determined by constant physiological parameters 10% of the carbon source in the medium was replaced with fully carbon labelled glucose ([U-13C]) for approximately 1.5 residence times, so that about 78% of the biomass was uniformly labelled. 2D [^13^*C*, ^1^*H*] COSY spectra of harvested and hydrolysed biomass were acquired for both aliphatic and aromatic resonances at 40°C on a Varian Inova 600 MHz NMR spectrometer. The software FCAL v.2.3.0 [19] was used to compute isotopomer constraints for 15 amino acids from the spectra. Detailed description of the cultivation set up can be found in [54] whereas similar ^13^*C *labeling set up, NMR experiments and spectral data analysis as were applied here have been described in [55].

In the second experiment *B. subtilis *was grown on shake flasks containing 50 ml M9 minimal medium. In the experiment, the medium was supplemented with 50 mg/L tryptophan and 3 g/L glucose labelled to the first carbon position ([1-13C]) (99%; Cambridge Isotope Laboratories) or a mixture of 0.6 g/L fully carbon labelled glucose ([U-13C]) (99%; Cambridge Isotope Laboratories) and 2.4 g/L unlabeled glucose as the sole carbon source. Fourteen derivatized amino acids were analyzed for ^13^*C *labeling patterns with a series 8000GC combined with an MD800 mass spectrometer (Fisons instruments). More information about the details of the measurement procedure can be found from [20] where identical measurement techniques were applied.

## Authors' contributions

AR, JR and EU are the main contributors of the computational methods given in the article. AR implemented the methods and ran the experiments. PJ constructed the models of metabolic networks and contributed to the development of the computational methods. NZ and HM contributed the development of the computational methods. AR, JR, NZ and PJ wrote the manuscript. All authors have read and accepted the contents of the manuscript.

## Supplementary Material

Additional file 1 **Model of central carbon metabolism of *S. cerevisiae *in SBML format**. Additional file 1 contains the stoichiometric model of central carbon metabolism of *S. cerevisiae *used in the experiments of the article. The model is provided as a text file containing SBML compliant descriptions of metabolites and reactions in the model. Metabolites are identified by their KEGG LIGAND codes. Click here for file

Additional file 2 **Illustration of the model of central carbon metabolism of ***S. cerevisiae*. Bidirectional reactions in PPP pathways are depicted as unidirectional for better readability. Click here for file

Additional file 3 **Model of central carbon metabolism of *B. subtilis *in SBML format**. Additional file 2 contains the stoichiometric model of central carbon metabolism of *B. subtilis *used in the experiments of the article. The model is provided as a text file containing SBML compliant descriptions of metabolites and reactions in the model. Metabolites are identified by their KEGG LIGAND codes. Click here for file

Additional file 4 **Illustration of the model of central carbon metabolism of *B. subtilis***. Bidirectional reactions in PPP pathways are depicted as unidirectional for better readability. Click here for file
