# Supplementary figures and images for: An analytic and systematic framework for estimating metabolic flux ratios from 13C tracer experiments
Source: BMC Bioinformatics. 2008 Jun 6;9:266. doi: 10.1186/1471-2105-9-266 (PMC2430715; doi:10.1186/1471-2105-9-266)

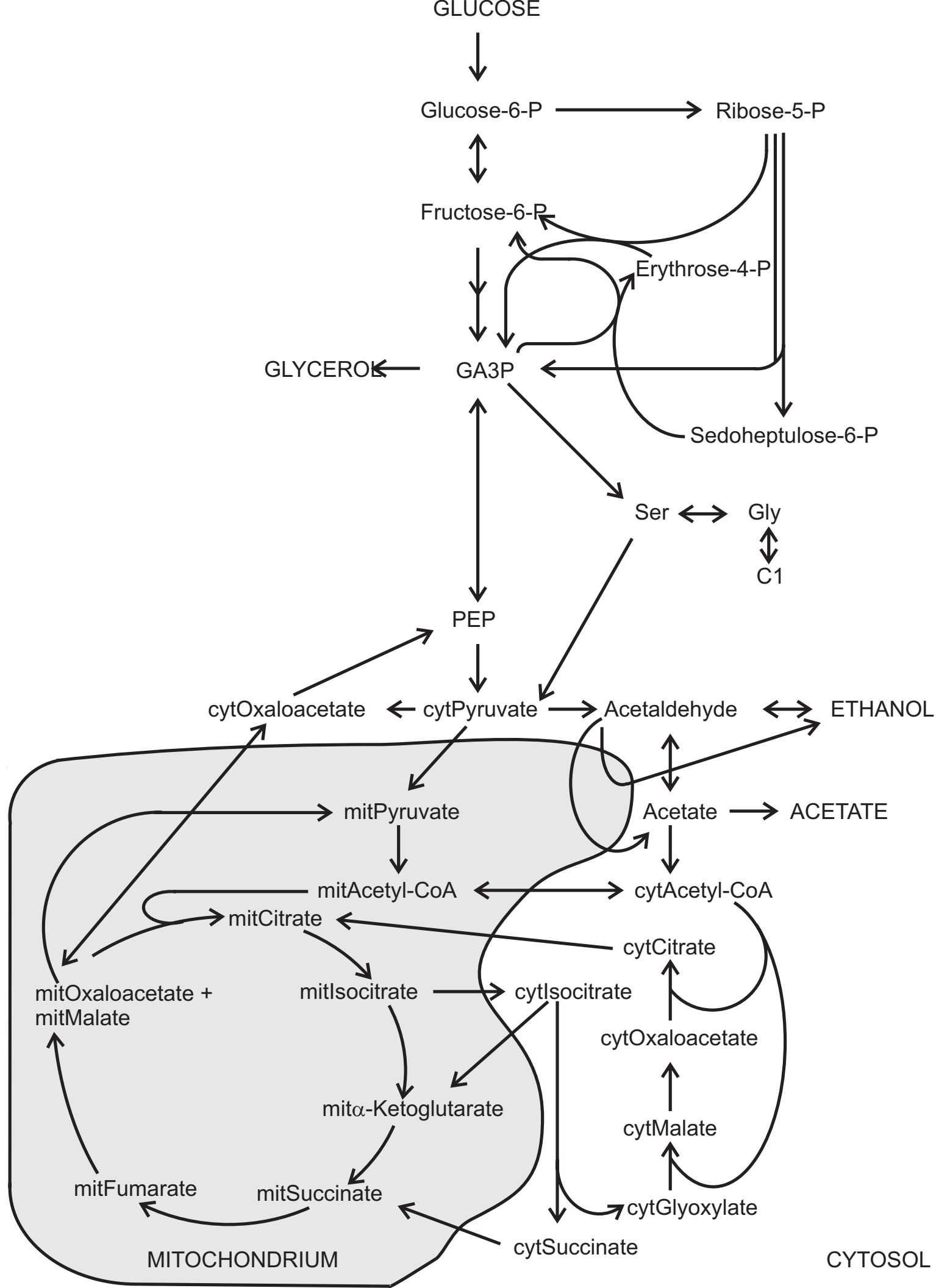

Supplement: Additional file 2 — Illustration of the model of central carbon metabolism of S. cerevisiae. Bidirectional reactions in PPP pathways are depicted as unidirectional for better readability. [file 1471-2105-9-266-S2.pdf]

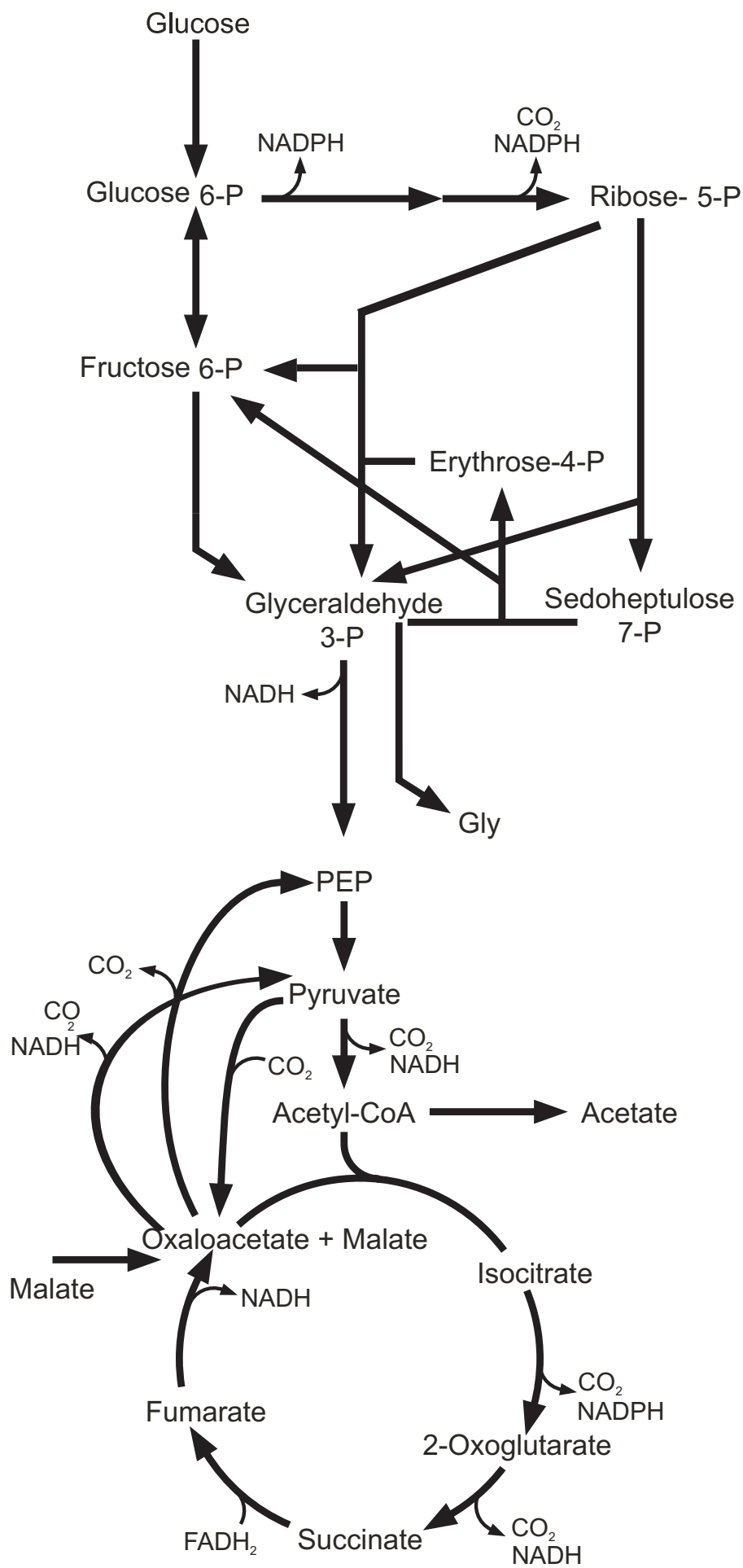

Supplement: Additional file 4 — Illustration of the model of central carbon metabolism of B. subtilis. Bidirectional reactions in PPP pathways are depicted as unidirectional for better readability. [file 1471-2105-9-266-S4.pdf]
